# The effect of CEO’s compensation in driving corporate ESG greenwashing: Evidence from China

**DOI:** 10.1371/journal.pone.0312247

**Published:** 2024-10-24

**Authors:** Kaile Li, Tzu-Yu Lin, Guifang Zhu

**Affiliations:** Accounting School, Nanfang College Guangzhou, Guangzhou, Guangdong, China; Zhejiang Sci-Tech University, CHINA

## Abstract

This study examines the relationship between CEO compensation schemes and ESG greenwashing behavior in Chinese listed firms during the period 2013–2022. We find that a CEO’s cash (equity) compensation has a significantly positive (negative) correlation with corporate ESG greenwashing behavior. From mechanism analysis, consistent with the agency problem view, firms engage in more severe ESG greenwashing behavior under a higher proportion of cash in the CEO compensation structure. Such distortion behavior is mitigated by higher internal control quality in firms having an equity incentive for their CEO under the convergence of interest viewpoint. Additional analysis reveals that corporates audited by large accounting firms and those with more media coverage exacerbate the positive correlation between CEO cash compensation and ESG greenwashing behavior, while government environmental regulations reinforce the inhibitory effect of CEO equity compensation on ESG greenwashing. Our results imply that different CEO compensation schemes can have opposite effects on limiting firms’ ESG greenwashing behavior in the Chinese context. Furthermore, we highlight that the question of form over substance principle to certain external governance mechanisms, leading CEO to exacerbate impression management of ESG disclosure.

## 1. Introduction

In recent decades, the environmental issue has emerged as one of the most significant economic and social challenges facing humanity. In accordance with the Greenhouse Gas Protocol, an enterprise is regarded as a principal reporting entity, necessitating the measurement and management of its carbon emissions. Consequently, it is apparent that an enterprise would experience increasing pressure from a range of stakeholders with regard to environmental sustainability. In light of the aforementioned considerations, fulfilling corporate social responsibility (CSR) has emerged as a crucial strategy in firms’ commercial practices, particularly in the context of environmental risk mitigation. Accordingly, the disclosure of environmental, social, and governance (ESG) information, regarding to the conduct of firms in a manner that confers significant benefits on stakeholders and society at large, has become a significant avenue for firms to demonstrate their capabilities in terms of CSR [1–3].

The qualitative disclosure of firms’ ESG activities is a common practice through various channels, including the annual report, sustainability report, ESG report, and official website, to present their commitment to social responsibility and sustainable development. Despite the existence of a series of principles for ESG disclosure frameworks set forth by the Global Reporting Initiative (GRI), controversy persists regarding the transparency of firms engaged in ESG activities. This is largely attributed to the lack of standardized guidelines and mandatory authentication for the preparation of ESG reporting. Accordingly, in accordance with the information asymmetry theory, firms are driven to exaggerate their ESG efforts. Specifically, firms may present themselves as more environmentally and socially responsible in their reports than they actually are. This phenomenon, referred to as ESG greenwashing, constitutes a form of deceptive ESG information dissemination.

Numerous empirical studies have explored factors influencing ESG greenwashing to mitigate this corporate misconduct, including national environmental quality policies [4], investor attention distraction [5], board gender diversity [6], larger board size [7], higher institutional ownership [7], and corporate transformation [8]. The extant literature indicates that executives bear the responsibility for the planning and promotion of corporate strategy [9, 10]. It is evident that ESG greenwashing can be defined as "strategic" ESG disclosure. Consequently, it is clear that CEOs are held responsible for the occurrence of such corporate misconduct [11]. Moreover, research has indicated that CEO compensation represents a significant motivating factor influencing their decision-making in the context of corporate strategic planning [12]. Nevertheless, there is still a paucity of knowledge regarding the relationship between CEO compensation schemes and ESG misconduct disclosure, particularly the incentive effect of either cash compensation or equity incentive. To address this gap in the literature, this study examines whether these incentives can mitigate CEOs’ misconduct, with a particular focus on the impact of CEO cash compensation (equity incentives) on ESG greenwashing in the Chinese context.

China provides an ideal setting for studying the association between executive compensation and ESG greenwashing at the corporate level. A 2018 statistical analysis by Willis Towers Watson revealed that equity compensation accounts for nearly 26% of total executive compensation in China A-share listed enterprises, compared to 55% in the US capital market. Equity incentives are not yet a widely used managerial tool in China. However, the 2018 amendment to the Company Law of the People’s Republic of China encourages listed firms to purchase tradable shares for CEO equity incentives and employee stock ownership plans (ESOPs).

This study examines the impact of different CEO compensation schemes on corporate ESG greenwashing using the data of China’s A-share listed companies from 2013 to 2022. The findings indicate that CEO cash compensation has a significantly exacerbating effect on ESG greenwashing, whereas CEO equity incentives have a mitigating effect on this misconduct. This suggests that ESG greenwashing is more pronounced in firms where CEO compensation is primarily in cash. CEOs with higher cash compensation are more likely to use ESG disclosures for self-promotion [13]. Conversely, based on agency theory, CEOs with higher equity compensation are more likely to prioritize stakeholder interests and avoid undeserved reputations. Considering the potential endogeneity problem, we employed instrumental variable (IV), propensity score matching (PSM) and system generalized method of moments (system GMM) estimation. Furthermore, the influence of external corporate governance mechanisms, including audit quality, media attention, and government environmental regulation, on this relationship is investigated. The findings indicate that elevated cash compensation is associated with increased ESG greenwashing, which is more prevalent in firms with financial reports audited by Big Four accounting firms or extensive media coverage. Conversely, higher equity compensation is linked to reduced ESG greenwashing, which is more pronounced in firms situated in regions with rigorous government environmental regulations.

The study makes a significant contribution to the existing literature on the subject by examining the impact of CEO compensation schemes, particularly cash compensation and equity incentives, on corporate behavior with regard to the disclosure of ESG information. The study makes three significant contributions to the existing literature. Firstly, this study contributes to the ongoing debate surrounding the incentive role of CEO compensation schemes from a corporate governance perspective. As an effective mechanism that may be used to directly drive managerial attention to specific objectives [10], a compensation scheme can, to a certain extent, be expected to mitigate the problem of agency conflicts in modern enterprises [14]. However, concerns remain due to inconsistent empirical results indicating that CEO may be motivated to take opposing managerial actions by different means of compensation schemes [12]. Consequently, within the context of Chinese institutional norms, our findings offer novel insights and substantiate a direct correlation between CEO equity incentives (cash compensation) and the curbing (or facilitation) of opportunistic managerial actions through ESG information misconduct, defined as ESG greenwashing. In comparison with studies conducted in developed countries (for example, the U.S. capital market), this finding is also consistent with the insight that long-term oriented compensation structures through equity incentives are more conducive to firms’ ESG practices.

Secondly, the study makes a significant contribution to the existing literature on the factors that influence the phenomenon of ESG greenwashing. While numerous studies have examined the macro-, firm-level, and CEO personality trait factors that influence ESG greenwashing [11], the literature on the effect of CEO compensation schemes remains limited and waiting for advanced investigation [14]. It is of particular importance to note that CEOs, who possess the authority to allocate resources within a firm through their decision-making capabilities, would be instrumental in impression management with regard to ESG greenwashing activities. Consequently, our study addresses this gap by examining the direct effect of CEO cash and equity compensation on the degree of ESG greenwashing.

Lastly, the study makes a contribution to the existing literature on the effect of CEO compensation schemes on corporate ESG greenwashing behavior by exploring the moderating effect of different external governance mechanisms in different contexts. The present study provides new evidence that the pressure of certain external governance mechanisms, particularly those audited by the Big Four or subject to media attention, on environmental issues leads to CEO engagement in opportunistic corporate ESG disclosure, as ESG greenwashing. However, the extent to which the curbing effect of CEO equity incentives on corporate ESG greenwashing can be more effectively achieved is contingent on the extent of environmental regulation by government supervisory authorities. Our findings present a contradictory evidence base in comparison with the existing literature on the disciplinary role of external governance mechanisms in the context of corporate misconduct.

The remainder of this study is organized as follows. Section 2 reviews the prior literature. Section 3 develops the corresponding hypotheses. Section 4 introduces the sample and methodology. Section 5 reports the main empirical results and robustness tests. Section 6 discusses further analysis, and Section 7 concludes the study.

## 2. Literature review

### 2.1 CEO compensation and corporate non-financial performance

Many variables related to CEOs, inspired by the upper echelon theory [15], have been incorporated into the study of sustainable corporate governance, of which one is CEO compensation. Stanwick and Stanwick (2001) [16] argue that the volatility of environmental reputation leads shareholders to not consider environmental reputation when designing compensation packages and to even view environmental expenses as an additional cost that should not be considered in a CEO’s strategic decisions. Thus, although an increase in CEO total compensation may lead to an improvement in a firm’s financial performance, it will reduce the firm’s environmental reputation. Using a sample of firms from 13 industrialized countries in Europe, Haque and Ntim (2020) [17] by contrast find that executive total compensation positively affects firms’ carbon performance, although this performance may be symbolic.

More scholars have conducted separate studies on CEO compensation in regard to cash compensation (short-term) and equity compensation (long-term) based on differences in pay structures [18, 19]. ESG disclosure may bring long-term benefits, so that when compensation is linked to shareholders’ returns, CEOs are better incentivized to substantively implement sustainability strategies and strive to improve environmental performance [20, 21]. Contrarily, cash compensation tends to be a short-term contract that can negatively impact corporate social responsibility (CSR) performance by inhibiting managerial risk-taking, exacerbating managerial myopia, and incurring short-term pressure for profitability [19, 22, 23].

The above conclusions are not widely shared, and in some cases, long-term stock and option-based compensation plans may not lead to higher environmental performance for firms. As Zou et al. (2015) [12] point out, due to the low demand for green products in emerging economies, the attitude of investors who do not care about environmental performance makes CEOs holding large amounts of stock only concerned with maximizing financial benefits, and the only way to spur agents’ interest in environmental causes is through high cash compensation. Fabrizi et al. (2014) [10], on the other hand, argue that monetary incentives (either cash or equity) significantly inhibit CSR compared to non-monetary monetary incentives. Kim and Kim (2023) [24] similarly note that excessive CEO compensation discourages firms from investing in ESG. In addition to this, Zhang and Zhang (2022) [25] find that executive compensation exhibits a U-shape threshold effect on corporate environmental responsibility performance and is positively influenced by industry competition.

### 2.2 Influencing factors of ESG greenwashing

The general use of greenwashing and even the academic debate on it seem to be broad and ambiguous. Some scholars consider greenwashing to be false information disseminated by firms in order to present an environmentally responsible public image [26]. A growing number of scholars defines it as a form of misleading communications, such as selective disclosure of positive information related to environmental or social performance [27], or positive communication about environmental information accompanied by poor environmental performance [28]. With ESG becoming an important criterion for evaluating firms’ non-financial performance, ESG disclosure turns out to be a new tool for corporate greenwashing [29]. Consistent with Yu et al. (2020) [7], in this study we define greenwashing as a firm’s attempt to manage stakeholders’ impressions by revealing a large amount of ESG data in order to obscure its less impressive overall ESG performance.

The factors that contribute to corporate greenwash are complex. Delmas and Burbano (2011) [28] group them into three levels: external, organizational, and individual. The actions of regulators, consumers, investors, as well as competitors may all be relevant to corporate greenwashing, except that the direction of this correlation has not been uniformly verified. Studies have found that financial inquiry letters as well as professional analysts exacerbate greenwashing [30, 31], whereas media attention, government regulation, and green finance work well as inhibitors [29, 32, 33]. Compared to research on the impact of external mechanisms, the relationship between internal corporate governance and greenwashing has not yet attracted enough attention from scholars, Zhang et al. (2023) [34] focus on the relationship between executive team characteristics and greenwashing, while more scholars use internal corporate factors as moderating or mediating variables in greenwash research [35, 36]. Executives play a central role in firms’ strategic and operational decisions, and this study enriches the literature on the intrinsic drivers of firms’ greenwashing by discussing the possible effects of different types of CEO compensation on greenwashing. In addition, studies generally agree that greenwashing is more harmful to society [37], although it may bring transient benefits to firms. Wu et al. (2020) [38] confirm that greenwashing also has a positive side by building a game-theoretic model of CSR investment, which misleads consumers’ purchasing decisions, but increases firms’ overall CSR spending.

## 3. Theoretical analysis and hypotheses’ development

### 3.1 CEO cash compensation and ESG greenwashing

Friedman (2007) [39] views CSR as an agency cost, arguing that managers waste shareholders’ wealth in pursuit of their own social mission, but in today’s business logic, the situation seems to be reversed. Over the past two decades, with the growth of environmental awareness and the popularization of the concept of sustainability on a global scale, investor interest has changed dramatically, and responsible investing, as represented by ESG, is no longer a niche phenomenon, with a large amount of money pouring into ESG products globally. Although there is no final agreement among scholars on the economic consequences of corporate ESG, most studies support the contribution of ESG activities to firm value, and there are various ways to realize value appreciation, such as wider financing channels [40], lower cost of capital [41], higher levels of innovation [42], and support from customers and employees [43]. In conclusion, the enlightened stakeholder theory has been widely confirmed in ESG practice.

News of corporate fudging on green initiatives is still commonplace, and one of the main reasons for this is that most of the positive impacts of ESG on companies take time to materialize, and the wait for long-term value undoubtedly exacerbates potential conflicts between managers and shareholders. Shareholders’ green awakening has led them to demand that CEOs do more for corporate sustainability—a demand that CEOs will not refuse from the standpoint of preserving their professional reputations, but cash compensation may break the tacit understanding between the two. Cash compensation for Chinese executives is directly linked to current profits [44], and in line with the cost-concerned perspective, incorporating green into a business strategy is a costly business practice that generates large cash outflows [45], which makes ambitious CEOs hesitant to invest substantively in ESG. In this context, greenwashing seems to be a shortcut to have your cake and eat it. In addition, CEOs need to justify their high cash compensation by bragging about their contributions to sustainability, so that shareholders feel they are contributing to the growth of the company.

In the absence of specific regulatory guidelines and audit endorsement of ESG data [7], CEOs can win more external resources through more aggressive ESG commitments while building up a good image of an environmental protection champion in front of shareholders and the external market. Conversely, symbolic ESG actions do not take up much cash flow and are less demanding on a CEO’s ability and energy. After all, reforming the organizational structure, greening production processes, and eco-innovation are not easy and are very challenging. As a result, the higher the CEO’s short-term compensation is, the more he or she will care about immediate benefits, the stronger the incentive to justify their pay, and be more inclined to maximize personal benefits by greenwashing. Based on the above analysis, this study proposes hypothesis 1.

H1: CEO cash compensation positively relates to ESG greenwashing.

### 3.2 CEO equity compensation and ESG greenwashing

The impact of equity incentives on firms has been controversial, and while most studies have concluded that equity-based compensation better aligns the interests of managers and shareholders than cash compensation [46], some studies have found that equity incentives increase the opportunistic tendencies of executives, and that CEOs with more equity are more concerned with short-term stock price fluctuations leading to short-sighted behaviors such as accounting manipulation [47]. Zou et al. (2015) [12] have argued that due to the low cost of environmental violations, negative environmental events of listed firms in China do not have a significant impact on stock market capitalization, and the financial market does not support firms’ environmental investments, so that firms with a higher percentage of executive shareholding perform worse in terms of environmental performance. Nevertheless, this study still expects equity incentives have a dampening effect on greenwashing in China.

Firstly, unlike relatively stable cash compensation, CEOs do not have to defend much of their personal wealth from the firm’s stock price, which reduces the incentive for CEOs to use greenwashing to justify their own equity compensation. Secondly, commendable ESG performance significantly reduces the likelihood of stock price crashes and increases firms’ cumulative abnormal returns during financial crises [48, 49]. It is clear that CEOs’ value-added gains from owning their own company’s stock can be used as an additional compensation for their implementation of ESG reforms. While greenwashing may also be used to drive up a firm’s stock price, stock market investors may be much less tolerant of failed ESG than consumers. Consumers who care about the environment may not necessarily buy greener products [50], and so even if a firm is found to display greenwash, the impact on their purchasing behavior is limited. For investors, however, once a company’s greenwashing behavior comes to light, the negative impact may no longer be limited to the environmental action itself, but may trigger a broader crisis of confidence in the form of concerns about the veracity of financial reporting, the quality of internal controls, and regulatory penalties, with a consequent decline in abnormal returns, and a backfire effect that may cause permanent damage to a firm’s reputation [51]. As a result, CEOs who are more sensitive to stock return volatility will more cautiously choose to utilize greenwashing for short-term gains due to higher equity compensation. In addition, the literature generally finds that equity incentives for executives stimulate their risk-taking [52]. This to some extent encourages CEOs to engage in green innovation and contributes to substantial ESG performance.

Compared to the study period of Zou et al. (2015) [12], the Chinese market has fundamentally changed its focus on sustainability in the last decade, with improving environmental regulations and growing scale of ESG investments in the capital market, so the potential risks associated with greenwashing can significantly harm the value of CEOs’ stock holdings. Zeng et al. (2023) [2] found that the implementation of equity incentives for executives in China is conducive to improving firms’ ESG performance, and Wu et al. (2022) [20] similarly confirms the effectiveness of executive equity incentive programs on green innovation in Chinese firms. Therefore, consistent with their views, this study proposes hypothesis 2.

H2: CEO equity compensation negatively relates to ESG greenwashing.

### 3.3 The effect of external monitoring mechanisms

Yue and Li (2023) [33] point out that external factors are key to motivating firms to greenwash, and scholars have been debating the role played by external monitoring mechanisms in corporate ESG endeavors. According to the neo-institutional theory, firms’ responses to institutional pressures are usually driven by two motivations: legitimization and efficiency. From the perspective of legitimization, firms may only symbolically comply with institutional pressures in order to gain organizational legitimacy, while from the perspective of economic efficiency, firms are more likely to engage in substantive initiatives related to environmental protection that can subsequently enhance their market value [17]. Both of these opposing behaviors have been confirmed and found under the effect of different types of external mechanisms.

Wang et al. (2022) [36] suggest that the media reduce information asymmetry through information dissemination, thereby inhibiting firms from corporate greenwashing, while Luo et al. (2022) [53] suggest that the media have a negative impact on firms’ environmental disclosure. Similarly, studies have argued that because Chinese firms are not substantially penalized for environmental misbehavior, they are less actively involved in improving environmental management [54], but Wang et al. (2022) [55] find in their study that environmental regulations serve as a good deterrent to firms’ pollution management and promote firms’ green technological innovation. Similar contradictory findings have been observed for the impact of investor concerns on corporate ESG performance [56, 57].

The role of high-quality auditing in promoting corporate ESG is likely to be more complex, and we do not have equal confidence in its ability to provide assurance on the quality of firms’ financial and non-financial information. Auditors can improve the quality of financial statements of firms with impaired ESG reputations and reduce the probability of restatement of their financial reports through increased audit efforts [58], but we still know very little about how auditors actually contribute to corporate sustainability. On the one hand, audit quality, as a key governance technique to address the agency problem of firms, can mitigate the opportunistic tendencies of managers and thus inhibit ESG greenwashing. On the other hand, greenwashing is not simply the dissemination of false information; it could also reflect optimism of a confident CEO in the ESG cohort effect, which is fueled by the fact that sensitivity to ESG disclosure on the part of auditors with advanced ideas increases a CEO’s ambitions and blindness to green endeavors.

There is evidence that firms have successfully coped with reputational crises by purchasing additional non-audit services from auditors after negative media coverage of ESG and have increased future market value as well as business performance [59]. While it is certainly good for firms that auditors have sufficient expertise to help them manage ESG risks, they may also be complicit in ESG greenwashing, either intentionally or unintentionally. The higher the CEO’s compensation is, the more attention will be attracted to these external mechanisms. Therefore, the following competing hypotheses are proposed.

H3a: External monitoring mechanisms positively moderates the relationship between CEO compensation and ESG greenwashing.
H3b: External monitoring mechanisms negatively moderates the relationship between CEO compensation and ESG greenwashing.

## 4. Data and research design

### 4.1 Sample and data

This study examines the impact of CEO compensation on ESG greenwashing in Chinese A-share listed firms from 2013 to 2022. Referring to other research [7, 29], we use Bloomberg ESG scores to measure the disclosure of firms’ ESG information. To reflect the actual ESG performance of firms, we use ESG data provided by Sino-Securities Index Information Service Company (Huazheng). Sino-Securities is a leading ESG information provider in China that focuses on the evaluation of ESG performance of China’s A-share and Hong Kong-listed companies. It has been widely used in empirical studies to measure the substantive ESG performance of Chinese firms by incorporating social responsibility indicators with Chinese characteristics, such as poverty alleviation and rural revitalization, based on the mainstream international methodology and practical experience [29, 31]. Except for the media attention variable from the CNRDS database, the other firm-level variables are from the CSMAR database.

Firms marked as having financial anomalies and delisting risks (ST or *ST) have been eliminated, leaving 8,028 firm-year observations in our sample. To reduce the effect of outliers, the main continuous variables are winsorized at the 1% and 99% levels.

### 4.2 Definition of variables

#### 4.2.1 Dependent variable: Greenwashing

As mentioned in Section 2.2, greenwashing in this study refers to the gap between a firm’s actual ESG performance and the content of its ESG disclosures. Huang et al. (2022) [60] measure corporate greenwashing using the content analysis method, but the coding process is inevitably affected by subjectivity of the individual researchers. Using questionnaires for greenwashing research [61, 62] can be unrepresentative due to sample size constraints and is more suitable for research targeting a specific industry. In contrast, using data from third-party rating agencies to measure corporate greenwashing is more objective [30]: First, because they cover a large enough sample size of firms, and second, because professional rating agencies have strict evaluation specifications and continuous support from a team of experts. The specific calculation appears as:

$$GW_{i,t}=\frac{ESG_{disi,t}-\bar{ESG_{disi,t}}}{\sigma ESG_{dis}}-\frac{ESG_{peri,t}-\bar{ESG_{peri,t}}}{\sigma ESG_{per}}$$ (1)

Here, *ESG*_*disi*,*t*_, measured by the Bloomberg ESG Disclosure Score, indicates the amount of ESG disclosed by firms to the public, and a higher Bloomberg ESG Disclosure Score indicates that firms disclose more non-financial information [7]. $\bar{{ESG}_{disi,t}}$ and *σESGdis* denote the mean and standard deviation of ESG disclosure in the same industry, respectively. *ESG*_*peri*,*t*_ denotes the actual performance of firms’ ESG, which is measured by the Sino-Securities (Huazheng) ESG Score and standardized in the same way as the Bloomberg ESG Disclosure Score. *GW*_*i*,*t*_ is the difference between the two, with larger values indicating a higher degree of greenwashing.

#### 4.2.2 Independent variable: CEO compensation

This study categorizes CEO compensation into cash (*P_cash*) and equity (*P_equity*). Cash compensation is the logarithm of the CEO’s annual cash compensation, which includes the CEO’s salary, bonuses, allowances, and other monetary compensation since listed firms in China are only required to disclose the total amount of executive compensation [63]. The implementation of equity incentives in China is still immature, and due to inherent data limitations, we refer to other research [64] and calculate equity pay based on the number of shares held by the CEO, specifically as the product of the number of shares held by a CEO as disclosed in annual reports and the average month-end share price during the year.

#### 4.2.3 Control variables

Following other research [29, 31], we identify a battery of control variables that likely correlate with ESG greenwashing, specifically firm size (*Size*), gearing ratio (*Lev*), investment opportunities (*Tbq*), return on assets (*Roa*), nature of property rights (*Soe*), the percentage of independent directors (*Indep*), and whether the CEO and board chair are the same person (*Dual*), which are used to measure firms’ levels of corporate governance. S1 Table provides a detailed definition of all the variables.

### 4.3 Regression models

In order to test the effect of CEO compensation on ESG greenwashing, this study constructs the following regression model:

$$GW_{i,t}=\alpha_{0}+\alpha_{1}P\_cash_{i,t-1}+\sum Controls_{i,t-1}+\sum Year+\sum Ind+\varepsilon$$ (2)

$$GW_{i,t}=\alpha_{0}+\alpha_{2}P\_equity_{i,t-1}+\sum Controls_{i,t-1}+\sum Year+\sum Ind+\varepsilon$$ (3)

Here, *GW*_*i*,*t*_ denotes the firm’s degree of greenwashing, and the core explanatory variables are *P_cash*_*i*,*t-1*_ and *P_equity*_*i*,*t-1*_, which denote the CEO’s annual cash compensation and equity compensation, respectively. According to H1, we expect that *α*_1_ should be positive, and the higher the CEO cash compensation is, the higher is the degree of greenwashing. On the contrary, *α*_2_ should be negative, and the CEO equity compensation can significantly inhibit ESG greenwashing.

In order to test the impact of external monitoring mechanisms, this study constructs model (4) and model (5):

$$\begin{array}{cc} GW_{i,t} & =\beta_{0}+\beta_{1}P\_cash_{i,t-1}+\beta_{2}Mod_{i,t-1}+\beta_{4}P\_cash_{i,t-1}\times Mod_{i,t-1}\\ & +\sum Controls_{i,t-1}+\sum Year+\sum Ind+\varepsilon \end{array}$$ (4)

$$\begin{array}{cc} GW_{i,t} & =\beta_{0}+\beta_{1}P\_equity_{i,t-1}+\beta_{2}Mod_{i,t-1}+\beta_{4}P\_equity_{i,t-1}\times Mod_{i,t-1}\\ & +\sum Controls_{i,t-1}+\sum Year+\sum Ind+\varepsilon \end{array}$$ (5)

Here, *Mod*_*i*,*t-1*_ denotes external governance variables, and this study selects audit quality (*Big4*), media attention (*Media*), and government environmental regulation (*Regu*) to represent audit monitoring, public monitoring and government monitoring, respectively, as defined in S1 Table. To mitigate potential endogeneity issues, we lag all independent variables by one period before conducting regression analyses. In addition, industry- and year-fixed effects are incorporated into the regression analysis. The regression analysis is also corrected for robust standard errors using individual firm-level clusters.

## 5. Empirical results

### 5.1 Descriptive statistics and correlation analysis

Table 1 lists the descriptive statistics of the main variables. The results show that the mean value of *GW* is -0.356, and the maximum and minimum values are 2.913 and -2.558, respectively. This indicates that the overall degree of greenwashing of firms in China is relatively low, which is basically consistent with the literature [29, 31]. The mean value of *P_cash* is 13.640, the maximum value is 15.840, the minimum value is 11.400, and the standard deviation is 0.836, which indicate that the cash compensation of CEOs in China’s listed firms is relatively concentrated, and the gap is relatively small. The mean value of *P_equity* is 7.593, the maximum value is 23.130, and the minimum value is 0, which denote that the equity compensation of CEOs varies greatly among China’s listed firms. They also verify that the equity incentives for executives have not been widely implemented in China [65]. Other control variables are basically consistent with the literature.

**Table 1:** Descriptive statistics.

Variable	Obs	Mean	Std.	Min	Max
*GW*	8,028	-0.356	1.098	-2.558	2.913
*P_cash*	8,028	13.640	0.836	11.400	15.840
*P_equity*	8,028	7.593	8.757	0.000	23.130
*Size*	8,028	23.240	1.285	20.540	27.010
*Lev*	8,028	0.484	0.195	0.077	0.869
*Tbq*	8,028	1.856	1.208	0.820	7.767
*Roa*	8,028	0.048	0.059	-0.152	0.234
*Dual*	8,003	0.196	0.397	0.000	1.000
*Indep*	8,028	0.374	0.054	0.313	0.571
*Soe*	7,869	0.533	0.499	0.000	1.000

S2 Table reports Pearson correlation coefficients among the variables, which shows that there is no significant multicollinearity problem between the variables.

### 5.2 Baseline regression

Table 2 shows the regression results of the direct impact of CEO compensation on ESG greenwashing. Based on the results in columns (1) and (2), we see that CEO cash compensation significantly and positively relates to ESG greenwashing without considering the control variables, while equity compensation shows a negative but not significant relationship with greenwashing. With the addition of control variables, the coefficient of *P_cash* remains positively related to *GW* at the 1% level, while the coefficient of *P_equity* also becomes significant at the 5% level. This result suggests after controlling for other factors that firms with higher CEO cash compensation are more inclined to greenwash, whereas equity compensation pushes CEOs’ long-term interests in sustainability to align with those of shareholders and reduces their tendency to greenwash, as verified by H1 and H2.

**Table 2:** Baseline regression results.

Variable	(1)	(2)	(3)	(4)
	*GW*	*GW*	*GW*	*GW*
*P_cash*	0.129***		0.083***	
	(4.34)		(2.94)	
*P_equity*		-0.003		-0.005**
		(-1.02)		(-2.05)
*Size*			0.185***	0.207***
			(7.16)	(8.08)
*Lev*			0.329**	0.328**
			(2.24)	(2.24)
*Tbq*			0.079***	0.084***
			(4.11)	(4.35)
*Roa*			-3.074***	-2.765***
			(-8.45)	(-7.62)
*Indep*			-1.077***	-1.159***
			(-3.02)	(-3.23)
*Soe*			-0.238***	-0.278***
			(-4.81)	(-5.53)
*Dual*			0.060	0.091*
			(1.15)	(1.72)
Constant	-2.115***	-0.335***	-5.436***	-4.743***
	(-5.25)	(-10.74)	(-8.45)	(-8.22)
Year & Industry	Yes	Yes	Yes	Yes
Obs	8,028	8,028	7,844	7,844
Adj *R*^2^	0.052	0.044	0.115	0.114

Notes: This table reports the main results, and the dependent variables are the variable *GW* in all specifications. In columns (1) and (2), we control for industry and year fixed effects and include no control variables. In columns (3) and (4), we additionally include control variables. The t-statistics are in parentheses.

***, **, and * represent significance at the 1%, 5%, and 10% levels, respectively.

The findings of this study refute the conclusions of Zou et al. (2015) [12], suggesting that after nearly a decade of development, the China’s market attitude towards sustainability has changed, especially in the context of the government’s commitment to reach a carbon peak before 2030 and achieve carbon neutrality before 2060, and that ESG investment is no longer an agency problem for firms, but rather a matter of greenwashing.

### 5.3 Robustness tests

In order to ensure the robustness of the benchmark regression results, we use a variety of methods to test the regression results in addition to lagging the dependent variable by one period in the regression model.

1. Model change. According to the definition of greenwashing in this study, when *GW* is greater than 0, it indicates that firms do more in ESG disclosure than in ESG actions, at which time there is greenwash. On the contrary, when *GW* is less than 0, there is no greenwash. Therefore, this study sets *Dummy_gw* as a dummy variable reflecting ESG greenwashing, which equals 1 when *GW* is greater than 0 and equals 0 otherwise, and then regresses Eqs (2) and (3) using logit model. Columns (1) and (2) of Table 3 show that the above findings remain robust.
2. Replacement of core explanatory variables. We use the annual cash compensation of executives (*Man_cash*) and whether the CEO holds shares (*Hold*) to replace *P_cash* and *P_equity*, respectively. *Man_cash* is measured as the natural logarithm of the average annual salaries and bonuses of the top management team in a firm. *Hold* is a dummy variable that equals 1 if the CEO holds the company’s shares in the year and 0 otherwise. The regression results are shown in columns (3) and (4) of Table 3, where we find that the stimulating effect of cash compensation and the dampening effect of equity compensation on greenwashing remain significant.
3. Add additional control variables. In order to minimize the impact of omitted variables, we further consider other possible influences in addition to models (1) and (2), such as whether the firm is loss-making (*Loss*), the size of the board of directors (*Board*), the age of the CEO (*Old*), as well as the degree of marketization (*Market*) and the degree of financing constraints (*Kz*), which reflect the external environment of the firm. The results in columns (5) and (6) of Table 3 show that the findings are still robust.

**Table 3:** Results from robustness tests.

Variable	(1)	(2)	(3)	(4)	(5)	(6)
	*Dummy_gw*	*Dummy_gw*	*GW*	*GW*	*GW*	*GW*
*P_cash*	0.128**				0.082***	
	(2.35)				(2.88)	
*P_equity*		-0.009*				-0.006**
		(-1.73)				(-2.19)
*Man_cash*			0.142***			
			(3.76)			
*Hold*				-0.095**		
				(-2.19)		
Constant	-9.950***	-8.969***	-5.857***	-4.747***	-4.881***	-4.489***
	(-8.43)	(-8.29)	(-8.78)	(-8.23)	(-5.50)	(-5.16)
Controls	Yes	Yes	Yes	Yes	Yes	Yes
Year & Industry	Yes	Yes	Yes	Yes	Yes	Yes
Obs	7,844	7,844	7,844	7,844	7,844	7,844
Adj / Pseudo R^2^	0.069	0.068	0.117	0.114	0.124	0.123

Notes: This table reports the results of robustness tests. In columns (1) and (2), we replace *GW* with a dummy variable and regress the main model using the logit model. As shown in columns (3) and (4), we replace the original independent variables with cash compensation that includes all executives and whether the CEO holds company stocks. Finally, we add several additional control variables on top of the original regression. In all columns, we control for industry and year fixed effects and include all control variables. The t/z-statistics are in parentheses.

***, **, and * represent significance at the 1%, 5%, and 10% levels, respectively.

### 5.4 Endogeneity issues

In order to effectively address the endogeneity problems caused by self-selection bias, omitted variables, and reverse causation, this study adopts the IV, PSM, Heckman and System GMM estimation to conduct the endogeneity test, respectively.

1. IV approach. Inspired by the literature [66, 67], we use two-period lagged CEO cash compensation (*L*.*P_cash*) and equity compensation (*L*.*P_equity*) as instrumental variables. Because compensation policies tend to have a certain degree of continuity, a CEO’s previous pay level may have some connection with current pay while not directly affecting ESG investment in the current year, satisfying the condition of exogeneity of the instrumental variables. Columns (1)-(4) of Table 4 present the regression results for the instrumental variables, and in the second-stage regression, *P_cash* (*P_equity*) significantly and positively (negatively) correlates with *GW* at the 5% level. The baseline regression results still hold. In addition, the test results of KP rk LM and KP rk Wald F indicate that the instrumental variables selected in this study are valid and there is no problem of unidentifiable and weak instrumental variables.
2. PSM approach. First, the sample is divided into experimental and control groups based on the industry median of CEO cash compensation and equity compensation. In the matching process, this study employs 1:1 nearest-neighbor matching while considering all the control variables discussed earlier. Columns (5)-(6) of Table 4 report the final results, and the benchmark regression results hold after controlling for differences in firm characteristics.
3. Heckman method. In the first stage, this study constructs dummy variables through the province-industry median of cash and equity compensation, which is 1 above the median and 0 otherwise. Inspired by Benlemlih et al. [21], we additionally introduce CEO’s age (*Old*) in the probit model of monetary compensation, and considering that equity incentives have distinct industry characteristics, we include the number of firms in the same province-industry with CEO’s shareholding (*Num*) as additional control variable in the first-stage model of equity incentives. The inverse Mills ratio (*Imr*) obtained in the first stage is carried into the second stage as an independent variable in the baseline models. The regression results are shown in columns (7)-(8) of Table 4 and support the H1 and H2.
4. System GMM model. Referring to Zhang and Yang (2023) [3], this study further employs the System GMM model, which mitigate endogeneity by using the lagged value of the dependent variable (*L*.*GW*). Columns (9)-(10) of Table 4 report the GMM results which are consistent with our main findings and satisfy the test conditions of the GMM model.

**Table 4:** Results from endogeneity tests.

Variable	IV Regression				PSM Model		Heckman		System-GMM	
	(1)	(2)	(3)	(4)	(5)	(6)	(7)	(8)	(9)	(10)
	First stage		Second stage							
	*P_cash*	*P_equity*	*GW*	*GW*	*GW*	*GW*	*GW*	*GW*	*GW*	*GW*
*L*.*P_cash*	0.719***									
	(0.02)									
*L*.*P_equity*		0.828***								
		(0.01)								
*L*.*GW*									0.552***	0.850***
									(7.96)	(7.13)
*P_cash*			0.099**		0.091***		0.139***		0.363**	
			(0.04)		(2.69)		(5.13)		(2.30)	
*P_equity*				-0.007**		-0.010***		-0.005**		-0.022**
				(0.00)		(-3.05)		(-2.35)		(-2.04)
*Imr*							1.185***	0.173*		
							(2.84)	(1.85)		
Constant	-	-	-	-	-4.878***	-4.494***	-10.223***	-4.689***	0.567	-6.014
					(-6.29)	(-5.70)	(-7.21)	(-13.85)	(0.17)	(-1.38)
Controls	Yes	Yes	Yes	Yes	Yes	Yes	Yes	Yes	Yes	Yes
Year & Industry	Yes	Yes	Yes	Yes	Yes	Yes	Yes	Yes	Yes	Yes
Obs	6,264	6,264	6,264	6,264	3,929	2,900	7,844	7,844	6,264	6,264
R^2^/Adj R^2^/ Wald chi^2^	/	/	0.083	0.081	0.104	0.114	462.14	876.12	/	/
KP rk LM	289.05***	512.94***								
KP rk Wald	2294.73	7284.12								
AR (1)									0.000	0.000
AR (2)									0.112	0.151
Hansen Test (p-valu)									0.100	0.201

Notes: This table reports the results of addressing endogeneity issues. The t-statistics are in parentheses.

***, **, and * represent significance at the 1%, 5%, and 10% levels, respectively.

### 5.5 Effect of external monitoring mechanisms

This study selects audit quality (*Big4*), media attention (*Media*), and government environmental regulation (*Regu*) as external monitoring variables, and models (4) and (5) are constructed to further explore the possible impacts of external mechanisms on the relationship between CEO pay and greenwashing in terms of audit monitoring, public monitoring and government monitoring. The results are shown in Table 5.

**Table 5:** Results from the moderating effect.

Variable	Mod = Big4		Mod = Media		Mod = Regu	
	(1)	(2)	(3)	(4)	(5)	(6)
	*GW*	*GW*	*GW*	*GW*	*GW*	*GW*
*P_cash*	0.030		0.069**		0.080***	
	(1.09)		(2.49)		(2.70)	
*P_equity*		-0.005**		-0.006**		-0.005
		(-1.98)		(-2.20)		(-1.64)
*Mod*	0.525***	0.638***	0.069***	0.085***	0.012	0.014
	(6.42)	(8.35)	(2.94)	(3.57)	(1.20)	(1.42)
*P_cash×Mod*	0.213***		0.050**		0.006	
	(3.10)		(2.18)		(0.75)	
*P_ equity×Mod*		0.017**		0.001		-0.001*
		(2.21)		(0.63)		(-1.73)
Constant	-3.314***	-3.088***	-4.691***	-4.135***	-5.456***	-4.716***
	(-5.24)	(-5.44)	(-7.42)	(-7.09)	(-8.05)	(-7.81)
Controls	Yes	Yes	Yes	Yes	Yes	Yes
Year & Industry	Yes	Yes	Yes	Yes	Yes	Yes
Obs	7,844	7,844	7,781	7,781	7,209	7,209
Adj R^2^	0.143	0.141	0.120	0.118	0.117	0.116

Notes: This table reflects the results on the moderating role of external mechanisms. In all columns, we control for industry and year fixed effects and include all control variables. The t-statistics are in parentheses.

***, **, and * represent significance at the 1%, 5%, and 10% levels, respectively.

Columns (1)-(2) of Table 5 show that high-quality auditing is significantly and positively associated with ESG greenwashing at the 1% level, and the coefficients of the interaction terms with CEO cash compensation and equity compensation are also all significantly positive. This suggests that superior auditors not only exacerbate ESG greenwashing behavior, but also strengthen the stimulus of cash compensation to greenwash while weakening the inhibitory effect of equity compensation on greenwashing. While most other studies have confirmed the positive significance of audit quality at improving firms’ ESG disclosure [68, 69], the findings in this study suggest that extensive disclosure may not necessarily lead to firms’ increased ESG transparency and that auditors should further broaden the examination of their substantive contributions to sustainability so as to enhance the credibility of ESG disclosure and alleviate other stakeholders’ concerns about corporate greenwashing.

The results in (3)-(4) show that the impact of media attention is basically consistent with audit quality. The only difference is that its weakening of the negative relationship between equity compensation and ESG greenwashing is not significant. Nevertheless, we still argue that media monitoring of firms does not play a role in identifying and suppressing greenwash, especially in the relationship between CEO cash compensation and greenwashing. This may be due to the pressure they invariably put on firms or due to their own lack of a deep understanding of ESG.

We finally find some comfort in the results in columns (5)-(6) of Table 5. Government environmental regulation does not directly and significantly relate to corporate greenwash, nor does it further strengthen the stimulus of CEO cash compensation to greenwashing. Moreover, the coefficient of its interaction term with equity compensation is significantly negative. It suggests that environmental regulation strengthens the inhibitory effect of equity compensation on greenwash, which to some extent validates the policy effectiveness of environmental regulation.

## 6. Further research

### 6.1 Mechanism analysis

Most CEO cash compensation in China is directly linked to short-term profits of the enterprise. As such, high cash compensation may be effective in motivating managers to improve the financial performance of the enterprise, but it will also lead to new agency problems when it comes to the sustainable development of the shareholders’ business. Through greenwashing, CEOs can establish a green image at the cost of less cash flow without having to consider any long-term pitfalls for shareholders, or even cause the firm to miss out on new opportunities for growth. A typical feature of greenwashing is to avoid long-term large capital expenditures and instead package itself through short-term expenditures. Therefore, drawing on other studies [70], ours examines the mechanism of the impact of CEO cash compensation on greenwashing by using a measure of agency cost (*Agency*), which is the ratio of administrative expenses to operating revenues after excluding executive cash compensation.

ESG initiatives require cross-functional collaboration within the organization, and high-quality internal control is an institutional guarantee for corporate ESG performance. Numerous studies have confirmed that effective internal control is crucial for managers’ accountability and the realization of corporate goals [71]. Therefore, it is reasonable to hypothesize that CEOs with higher equity compensation will ensure the quality of ESG implementation by establishing a sound internal control system, which in turn inhibits the tendency of greenwashing. In this study, we set up a dummy variable *IC* to measure the quality of firms’ internal control, which equals 1 when internal control is not deficient and 0 otherwise.

Referring to the mediating effect test model proposed by Baron and Kenny (1986) [72], this study examines the possible paths of influence between CEO pay and greenwash by constructing model (6) and model (7) based on model (2) and (3). Here, where *Med*_*i*,*t*_ is the mediating variable that denotes the agency cost (*Agency*) and the quality of internal control (*IC*), respectively. In addition, we retain previous control variables in the regression process and conduct validity tests using Sobel’s test and the Bootstrap approach with 1,000 repetitive samplings.

Table 6 demonstrates the regression results. Columns (1)-(3) indicate that CEO cash compensation exacerbates firms’ agency costs and leads to greenwashing through partial mediation effects. In column (5), the coefficient of CEO equity compensation is significantly positive at the 5% level, indicating that the higher the CEO equity compensation is, the more importance is attached to the construction of a firm’s internal control, and a properly established internal control mechanism can help the firm to improve the science of decision-making and enhance the reliability of information disclosure. Column (6) shows that effective internal control significantly reduces the degree of firms’ greenwashing, while playing a partial mediating role in the inhibiting effect of CEO equity compensation on greenwashing.


$$Med_{i,t}=\lambda_{0}+\lambda_{1}P\_cash_{i,t}/P\_equity_{i,t}+\sum Controls_{i,t}+\sum Year+\sum Ind+\varepsilon$$ (6)

$$\begin{array}{cc} GW_{i,t} & =\delta_{0}+\delta_{1}P\_cash_{i,t-1}/P\_equity_{i,t-1}+\delta_{2}Med_{i,t-1}+\sum Controls_{i,t-1}\\ & +\sum Year+\sum Ind+\varepsilon \end{array}$$ (7)

**Table 6:** Mechanism tests: Agency cost and internal control.

Variable	Med = Agency			Med = IC		
	(1)	(2)	(3)	(4)	(5)	(6)
	*GW*	*Med*	*GW*	*GW*	*Med*	*GW*
*P_cash*	0.083***	0.002**	0.082***			
	(2.94)	(1.98)	(2.89)			
*P_equity*				-0.005**	0.014**	-0.005*
				(-2.05)	(2.41)	(-1.86)
*Med*			0.767*			-0.093**
			(1.88)			(-2.32)
Constant	-5.436***	0.198***	-5.588***	-4.743***	5.296***	-4.595***
	(-8.45)	(7.93)	(-8.50)	(-8.22)	(4.63)	(-7.81)
Controls	Yes	Yes	Yes	Yes	Yes	Yes
Year & Industry	Yes	Yes	Yes	Yes	Yes	Yes
Obs	7,844	7,844	7,844	7,844	7,506	7,506
Adj / Pseudo *R*^2^	0.115	0.396	0.116	0.114	0.115	0.115
Sobel	z = 2.211**			z = -3.142***		
Bootstrap (1000)	z = 2.240**			z = -3.17***		

Notes: This table reports the results of mechanism tests. Columns (2) and (3) incorporate the proxy for agency costs as the mediating variables, which is the ratio of management expenses after excluding executives’ cash compensation to total assets. Columns (5) and (6) incorporate whether there are defects in internal control as a proxy for the quality of internal control. In all columns, we control for industry and year fixed effects and include all control variables. The t/z-statistics are in parentheses.

***, **, and * represent significance at the 1%, 5%, and 10% levels, respectively.

### 6.2 Heterogeneity analysis

#### 6.2.1 Industry heterogeneity

Prior research has indicated that high pollution or high carbon-emitting firms have stronger incentives to participate in greenwashing than other firms. On the one hand, high pollution firms receive more social attention because they are direct producers and emitters of various hazardous substances. As CEOs of such firms bear greater pressure for organizational legitimacy, they are more inclined to emphasize the social value of their companies through selective disclosure of ESG information. On the other hand, subject to policy requirements, high pollution firms need to increase their investment in green production and industrial upgrading, either voluntarily or involuntarily, and the additional expenditure gives CEOs enjoying high compensation stronger incentives to utilize greenwashing to justify their pay. Based on the above analysis, we expect that internal agency problems arising from cash compensation are more severe in high pollution firms than in other firms, while the governance effect of equity compensation is weakened by external pressures.

This study divides the sample into two groups according to whether the firms belong to the high pollution industry (*pollu*) or not, and the regression results appear in Table 7. Columns (1)-(2) show that CEO cash compensation is significantly and positively related to corporate greenwashing in both high and low pollution firms, but the stimulus is greater in high pollution firms. The results in columns (3)-(4) indicate that the inhibitory effect of CEO equity compensation on greenwashing is significantly effective only in low pollution firms. These findings reconfirm that high pollution firms are more likely to engage in greenwashing than other firms from the perspective of CEO compensation.

**Table 7:** Heterogeneity of industry.

Variable	*GW*			
	(1)	(2)	(3)	(4)
	High pollution	Low pollution	High pollution	Low pollution
*P_cash*	0.093*	0.074**		
	(1.86)	(2.17)		
*P_equity*			-0.004	-0.006*
			(-0.73)	(-1.87)
Constant	-6.004***	-5.289***	-5.174***	-4.724***
	(-5.28)	(-6.78)	(-5.10)	(-6.72)
Controls	Yes	Yes	Yes	Yes
Year & Industry	Yes	Yes	Yes	Yes
Obs	2,494	5,350	2,494	5,350
Adj *R*^2^	0.140	0.107	0.138	0.106
FP test	-0.019		-0.002	
p value	0.071*		0.039**	

Notes: This table reports the results of subsample regression divided by the heavily-polluted firms. In all columns, we control for industry and year fixed effects and include all control variables. The t-statistics are in parentheses. The penultimate row presents the coefficient difference (b0-b1) after the Fisher’s Permutation test (bootstrap 1,000 times).

***, **, and * represent significant at 1%, 5%, and 10% levels, respective.

#### 6.2.2 Operational risk heterogeneity

Companies with good financial status are better able and willing to engage in environmental and social practices that respond to wider stakeholder demands, in contrast, companies with financial shortfalls and precarious profitability tend to prioritize short-term financial goals [73]. Chams et al. (2021) [74] state that financial achievements are perceived as pre-requisites or antecedents of ESG adoption. As a result, firms’ operational risk distracts the CEO from the environmental cause and also increases the negative reaction of shareholders to the firm’s environmentally friendly practices, at which point it is no longer wise for CEOs to attempt to use greenwashing to justify their own compensation, and they may even maintain green hushing to win the trust of shareholders as well as the board of directors.

In this study, we use cash flow volatility to measure firms’ operational risk (*Risk*), the results in Table 8 basically validate our hypothesis. Columns (1)-(2) show that the stimulus effect of cash compensation on greenwashing is no longer significant when firms face higher operational risks, and that the instability of firms’ operations distracts CEOs from greenwashing and weakens the incentives for CEOs to use it to justify their own cash compensation. In addition, although the subgroup regressions of equity compensation on greenwashing do not pass the between-group variation test in columns (3)-(4), the fact that the inhibitory effect of equity compensation on greenwashing is significant only among firms with higher operational risk also supports our conclusion to some extent.

**Table 8:** Heterogeneity of operational risk.

Variable	*GW*			
	(1)	(2)	(3)	(4)
	High risk	Low risk	High risk	Low risk
*P_cash*	0.026	0.145***		
	(0.80)	(3.92)		
*P_equity*			-0.006*	-0.005
			(-1.91)	(-1.31)
Constant	-4.291***	-6.459***	-4.087***	-5.178***
	(-5.72)	(-7.84)	(-5.84)	(-7.18)
Controls	Yes	Yes	Yes	Yes
Year & Industry	Yes	Yes	Yes	Yes
Obs	3,979	3,865	3,979	3,865
Adj *R*^2^	0.105	0.140	0.106	0.133
FP test	0.119		0.002	
p value	0.000***		0.171	

Notes: This table reports the results of subsample regression divided by operational risk, which is measured by the volatility of net cash flows from operating activities. Specifically, we rolling calculate the standard deviation of cash flows every 3 years (t-2, t-1, t) and then use the industry median as the basis for subsamples. In all columns, we control for industry and year fixed effects and include all control variables. The t-statistics are in parentheses. The penultimate row presents the coefficient difference (b0-b1) after the Fisher’s Permutation test (bootstrap 1,000 times).

***, **, and * represent significant at 1%, 5%, and 10% levels, respective.

## 7. Conclusions

With the increasing attention paid to corporate ESG factors from all walks of life, the phenomenon of greenwashing has spread from the earliest consumer goods market to ESG disclosure or sustainability reporting, sparking the market’s concern and vigilance. This study empirically analyzes the impact of CEO compensation on ESG greenwashing using data from A-share listed companies in China from 2013–2022. Findings show that different forms of CEO pay exhibit different roles in promoting corporate ESG endeavors, and in general, the higher the CEO cash compensation is, the greater is the degree of ESG greenwashing. On the contrary, the higher the equity compensation held by the CEO is, the lower is the degree of ESG greenwashing. This finding is also consistent with the insight provided by the study of Kim and Kim [24] regarding the U.S. capital market, namely that long-term oriented compensation structures are more conducive to firms’ ESG practices. In addition, this study finds that high-quality audits reinforce the greenwash stimulus of cash compensation while weakening the disincentive effect of equity compensation. Media attention has a similar effect, although it does not significantly affect the relationship between equity compensation and greenwashing. Moreover, local government environmental regulations reinforce the disincentive effect of equity compensation and do not further exacerbate the greenwashing effect of cash compensation.

The findings herein suggest in China’s current market that soft external monitoring has a weak deterrent effect in the area of greenwashing and even puts invisible pressure on managers to exacerbate greenwashing, while hard environmental regulations play a positive role in greenwashing monitoring. Further analysis reveals that CEO cash compensation increases firms’ agency costs and thus stimulates greenwashing, while CEOs with equity compensation curb their tendency to greenwash by improving internal controls. A heterogeneity test finds that the effect of cash compensation on greenwashing is greater in firms with high pollution and lower operational risk, while the inhibitory effect of equity compensation mainly exists in the rest of the firm sample.

This study not only systematically reveals the internal motives behind ESG greenwashing from the perspective of CEO compensation, but also provides useful insights for listed companies to optimize compensation design and external supervisory authorities to strengthen the management of corporate ESG disclosure. First, corporate shareholders should recognize that the form of CEO compensation is crucial, and that credible ESG investment is necessary for the long-term development of corporations under the trend of sustainable development. Firms should mitigate agency conflicts with CEOs and guide them to value long-term corporate value through rational compensation structure design, thus avoiding the potential risks that greenwashing brings to firms.

Second, the roles of auditors and the media in the field of ESG greenwashing need to be strengthened. They are not necessarily being accused of intentionally helping firms to go greenwash, but rather excellent auditors and the media’s extensive promotion of the concept of sustainability are pushing more CEOs to awaken to the benefits of greenness and to recognize the importance of ESG so as to make relevant information disclosures. In any case, auditors and the media should further improve their professional skills in order to do more in monitoring the substantive green actions of enterprises.

Finally, formal government environmental regulations are effective in suppressing ESG greenwashing. In the future, local government departments should appropriately increase the intensity of existing regulations in a gradual and orderly manner and combine them with diversified institutional means. Doing so can help further realize the green governance effect of environmental regulations.

## Supporting information

S1 Table Definition of variables. (DOCX)

S2 Table Pearson correlation matrix of the main variables. (DOCX)

S1 Data (XLSX)
