# Supplementary material for: The effect of CEO’s compensation in driving corporate ESG greenwashing: Evidence from China
Source: PLoS One. 2024 Oct 24;19(10):e0312247. doi: 10.1371/journal.pone.0312247 (PMC11500961; doi:10.1371/journal.pone.0312247)
Supplement: S1 Table — (DOCX) [file pone.0312247.s001.docx]

**Supporting Information**

**S1 Table. Definition of variables.**

| **Variable** | **Definition** |
| --- | --- |
| *GW* | Gap between firms’ ESG disclosure scores and actual ESG performance as modeled by (1) |
| *P_cash* | Logarithm of the CEO’s cash compensation for the year, including salaries, bonuses, allowances, and other monetary-based compensation |
| *P_equity* | Logarithm of the CEO’s equity compensation for the year, from the product of the number of shares held at the end of the year and the average share price at the end of the year |
| *Size* | Logarithm of total assets |
| *Lev* | Total liabilities/total assets |
| *Tbq* | Tobin’s Q |
| *Roa* | Return on assets; net income divided by average net assets |
| *Indep* | Percentage of independent board members |
| *Dual* | Dummy variable that equals 1 if CEO and Chair are the same person |
| *Soe* | Dummy variable that equals 1 if the firm is state-owned and 0 otherwise |
| *Big4* | Dummy variable that equals 1 if the firm is audited by an international Big-4 audit firm and 0 otherwise. |
| *Media* | Ln (annual media coverage of the company+1) |
| *Regu* | Ln (number of administrative penalties for environmental protection at the prefecture level+1) |
