# Supplementary material for: The effect of CEO’s compensation in driving corporate ESG greenwashing: Evidence from China
Source: PLoS One. 2024 Oct 24;19(10):e0312247. doi: 10.1371/journal.pone.0312247 (PMC11500961; doi:10.1371/journal.pone.0312247)
Supplement: S2 Table — (DOCX) [file pone.0312247.s002.docx]

**Supporting Information**

**Table2. Pearson correlation matrix of the main variables.**

|  | (1) | (2) | (3) | (4) | (5) | (6) | (7) | (8) | (9) | (10) |
| --- | --- | --- | --- | --- | --- | --- | --- | --- | --- | --- |
| (1) *GW* | 1.000 |  |  |  |  |  |  |  |  |  |
| (2) *P_cash* | 0.084^***^ | 1.000 |  |  |  |  |  |  |  |  |
| (3) *P_equity* | -0.038^***^ | 0.232^***^ | 1.000 |  |  |  |  |  |  |  |
| (4) *Size* | 0.184^***^ | 0.305^***^ | -0.074^***^ | 1.000 |  |  |  |  |  |  |
| (5) *Lev* | 0.153^***^ | 0.046^***^ | -0.145^***^ | 0.497^***^ | 1.000 |  |  |  |  |  |
| (6) *Tbq* | -0.048^***^ | 0.025^**^ | 0.173^***^ | -0.414^***^ | -0.385^***^ | 1.000 |  |  |  |  |
| (7) *Roa* | -0.139^***^ | 0.210^***^ | 0.226^***^ | -0.103^***^ | -0.463^***^ | 0.368^***^ | 1.000 |  |  |  |
| (8) *Dual* | 0.019^*^ | 0.115^***^ | 0.277^***^ | -0.089^***^ | -0.089^***^ | 0.106^***^ | 0.112^***^ | 1.000 |  |  |
| (9) *Indep* | -0.038^***^ | 0.019^*^ | -0.006 | 0.084^***^ | 0.032^***^ | 0.019^*^ | 0.005 | 0.090^***^ | 1.000 |  |
| (10) *Soe* | -0.025^**^ | -0.152^***^ | -0.376^***^ | 0.249^***^ | 0.196^***^ | -0.189^***^ | -0.201^***^ | -0.291^***^ | 0.005 | 1.000 |
| Note: ***, **, and * represent significance at the 1%, 5%, and 10% levels, respectively | | | | | | | | | | |
